# What was the impact of a global pandemic (COVID-19) lockdown period on experiences within an eating disorder service? A service evaluation of the views of patients, parents/carers and staff

**DOI:** 10.1186/s40337-021-00368-x

**Published:** 2021-01-19

**Authors:** Hannah Shaw, Sarah Robertson, Nadia Ranceva

**Affiliations:** 1 The Eating Disorder Young Person’s Service, Alder Hey Children’s Hospital Foundation Trust, Liverpool, UK; 2 Royal Holloway University of London, Egham, UK; 3 Vilnius University, Vilnius, Lithuania

**Keywords:** COVID-19, Eating disorders, Global pandemic, Lockdown, Parent/Carer experience, Patient experience, Service development, Service evaluation, Service satisfaction, Staff experience

## Abstract

**Background:**

The World Health Organization declared the outbreak of COVID-19 as a global pandemic on the 11th March 2020. As a result, the UK Government imposed severe restrictions on working and social contact as part of “lockdown.” Whilst the full extent of the pandemic’s impact on eating disorder patients is unknown, the literature suggests that patients with pre-existing mental illness may be more vulnerable to the mental health impacts. In addition, the restrictions greatly reduced the access to mental health services and presented new challenges to service delivery. A service evaluation was carried out to explore how the COVID-19 global pandemic changed service provision in a young person’s eating disorder service and how this affected patient, family and staff experiences.

**Methods:**

An audit was carried out to explore how the lockdown period had impacted referrals and service delivery. Quantitative data was collected in an online survey and qualitative data was collected in two formats: open ended answers as part of the online survey and open-ended focus groups, structured using narrative enquiry. The 43 participants consisted of 12 patients, 19 parents/carers, and 12 staff members. Patients were under the age of 18 and had a diagnosis of an eating disorder.

**Results:**

COVID-19 and lockdown increased the pressure on the service and changed service provision significantly. This has impacted the relational experiences for patients and their carers and staff have been faced with new challenges. Patients, parents/carers and staff all preferred face-to-face appointments over virtual options. There was no difference in service satisfaction before and during COVID-19.

**Conclusions:**

It is possible to provide an eating disorder service in lockdown restrictions that patients and parents report high satisfaction with. Providing face-to-face appointments at the beginning of treatment and including families in the planning should be prioritised. Staff support is crucial to be able to continue delivering high quality services. The key themes are identified, and clinical recommendations are made to guide service delivery.

## Plain English summary

Online survey’s and focus groups were used to find out how the COVID-19 global pandemic changed how support was provided by a young person’s eating disorder service. We were interested in finding out how these changes and new ways of working were experienced by patients, families and staff. The aim was to find out how best to use the new methods to meet the needs of those accessing the service and those working for the service. Staff told us that there was an increased pressure on the service during COVID-19. We found out that patients, families and staff all preferred face-to-face appointments over virtual options (e.g. telephone calls or video calls). Although all participants told us there were significant changes to the service provided, there was no difference between the service satisfaction before and during COVID-19. This suggests that it is possible to provide an eating disorder service that patients, their families and staff are satisfied with in lockdown restrictions. However, staff must be receiving the right level of support for this to continue. Additionally, the feedback suggests that services should provide face-to-face appointments at the beginning of treatment and must ensure families are involved in the care and planning.

## Background

The World Health Organization declared the outbreak of COVID-19 as a global pandemic on the 11th March 2020. On March 23rd, 2020, the UK Government imposed severe restrictions on working and social contact as part of “lockdown”, which greatly reduced the access to health services, and presented new challenges to service delivery.

As a result, there are rising concerns about the detrimental impact of the pandemic on mental health [7]. Studies have found that mental health in the UK has deteriorated compared with pre COVID-19 trends [18]. The literature suggests that patients with pre-existing mental illness may be more vulnerable to the mental health impacts and at higher risk of relapse or new episodes of their disorder [20, 21]. Whilst the full extent of the pandemic’s impact on eating disorder (ED) patients is unknown, emerging evidence suggests there will be a detrimental effect [2].

As a result of COVID-19, many healthcare services are only offering urgent visits and inpatient treatment for severe ED cases. Restrictions have led to the closure of day hospitals and outpatient facilities across the world and where possible, online/virtual treatments have been recommended [6]. While online and e-health measures have been developed for EDs, more research is needed to understand how they are experienced, and how to implement them effectively [1]. Patients and families will be burdened with more responsibility and self-management due to the lack of face-to-face accountability (e.g. no/less regular weigh-ins) with professionals [6]. There are also concerns that video calls could be a triggering form of communication due to the heightened awareness of bodily self which could prompt self-criticism harmful to recovery [6]. Moreover, we are yet to find out how these new ways of working impact the staff within ED services.

When lockdown restrictions were imposed, the Eating Disorder Young Person’s Service (EDYS) at Alder Hey Children’s Hospital had to quickly adjust to new ways of working. In order to reduce the number of patients attending the clinic, the community nursing team were engaged to carry out physical observations (lying and standing blood pressure, pulse and weight) at patients’ homes. Results were then emailed to the responsible clinicians. As more infection control measures were implemented onsite, the service opened a physical monitoring clinic, where patients would come in for a 15-min physical observation appointment. All other appointments were offered virtually. Clinicians reported that patients were not responding as well to treatment when delivered in this way and as a result both mental and physical health of many patients was deteriorating. Therefore, the decision was made to reintroduce face-to-face appointments for patients deemed as high-risk in June and July.

It is crucial that we continue to support the ongoing provision of vital mental health services, whilst seeking to mitigate the negative impact of restrictions on vulnerable groups [11]. In order to understand the best way to do this, we conducted a mixed methods service evaluation aimed at exploring the impact of the pandemic on patients, family and staff experiences. This service evaluation was conducted in the EDYS at Alder Hey Children’s NHS Foundation Trust.

## Methods

An audit was carried out using data from the Electronic Patient Record System used by the trust to gain an understanding of how the lockdown period had impacted referrals and service delivery. A mixed method design was used to conduct a service evaluation aimed at understanding how the eating disorder service changed, and how that was experienced by staff, family, and patients. Quantitative data was collected using an online survey containing a series of questions using a five-point Likert scale ranging from strongly disagree (1) to strongly agree (5). Qualitative data was collected in two formats: open ended answers as part of the online survey and open-ended focus groups, structured using narrative inquiry.

### Participants

43 participants opted to take part: 12 patients, 19 parents/carers, and 12 members of staff. All participants had experience of the service during COVID-19. As participation was based on availability, no power analysis was conducted to inform sample size. No demographic information was collected to ensure responses remained anonymous. All patients were under the age of 18 and had a diagnosis of typical or non-typical anorexia nervosa (AN), bulimia nervosa (BN) or binge-eating disorder (BED).

### Procedure

This service evaluation was approved by the Clinical Audit Department at Alder Hey Children’s NHS Foundation Trust. We sought consent from all participants at the start of the survey in the form of a tick box question and verbally at the beginning of each focus group.

### Quantitative

We created a survey which was informed by discussions with service users at CAMHS (Child and Adolescent Mental Health Service) participation groups. Based on this feedback, we adapted the existing measure of CAMHS service satisfaction (Experience of Service Questionnaire, ESQ), recommended by CORC (Child Outcome Research Consortium). The survey consisted of two sections: experience of service during COVID-19, and experience of service before COVID-19. Only participants who stated they had been involved with the service before COVID-19 were taken to this section of the survey (*n* = 29).

We sent a short explanation about the service evaluation and the link for the relevant survey, to patients and parent/carers via text to registered numbers on the hospital record system. This was sent to staff via email.

### Qualitative

We then conducted open ended focus groups structured using narrative inquiry. Narrative research begins with the premise that we tell stories about our lives [17]. Narrative interviews involve two parts: 1) an open ended exploratory question; e.g., “*what has it been like for you receiving a service/providing a service in COVID-19?*”; 2) follow up prompts based on what participants describe, in the order they describe it; e.g. “*you said that … what did it feel like for you in the moment*?”, “*you mentioned … can you tell me more about what happened*?” [19]. Using this approach, we conducted three individual focus groups with groups of staff, parents, and patients. Staff attended the focus group prior to an MDT team meeting. We texted parents and patients an invitation to join the focus groups via MS teams. Focus groups were recorded and anonymously transcribed.

### Analysis

#### Quantitative

We used Excel for descriptive statistics and JASP for inferential statistical analysis. Due to the non-parametric nature of the data, we used a Wilcoxon Signed-Rank test to determine whether there was significant difference between service satisfaction before COVID-19 and during COVID-19. Patient, parent/carer and staff scores were collated to carry out the statistical analysis as a result of the small individual sample sizes which would have led to very low power for detecting group differences.

#### Qualitative

Narrative analysis provides a vantage point that allows others to analyse how the storyteller constructs meaning. Storytellers depict how characters, events, interactions, and outcomes are related [13, 16]. Informed by structural and thematic traditions of narrative inquiry, we listened back to stories shared in focus groups and mapped out the key events described, and how they were experienced, into a table for patients, parents/carers and staff. The qualitative feedback received through the Qualtrics survey was also mapped into this process and overarching themes were identified through thematic analysis [3].

## Results

### How the lockdown period impacted the EDYS referrals and service delivery

Table 1 shows the number of referrals accepted by EDYS throughout the same time period (March – July) in 2019, when there were no lockdown restrictions, and 2020, during the initial lockdown period. Although less referrals were accepted, more of these were triaged as urgent, meaning that patients presentations were more acute. Thus, patients had worse ED symptomology, physical health observations that were further away from the normal range and higher scores on risk assessments. There was also a marked increase in the number of admissions of ED patients to the general peadiatric ward; meaning that patients were too physically and mentally unwell to remain at home.

**Table 1:** Number of referrals accepted by EDYS from March to July in 2019 and 2020

	March – July 2019		March – July 2020	
Accepted Referrals	n	%	n	%
Urgent	7	20.59	10	34.48
Routine	27	79.41	19	65.52
Total	34	100.00	29	100.00
Paediatric Admissions	4		9	

More recent data collected by the service (Table 2) shows that more referrals were accepted in 2020 than in 2019 over the same time period (1st January - 2nd December). It also shows that the percentage increase was larger for urgent referrals (150%) than routine referrals (129%).

**Table 2:** Number of referrals accepted by EDYS from 1st January – 2nd December in 2019 and 2020 split by type

Year	Urgent (n)	Routine (n)	Total (n)
2019	14	107	121
2020	21	138	159
Increase in accepted referrals (%)	150	129	132

Table 3 presents the number of face-to-face and virtual appointments offered by EDYS each month from December 2019 to July 2020. The data shows that on average there was an overall increase in appointments offered during lockdown (M = 402.4, SD = 37.90) compared to previous months (M = 265, SD = 37.27).

**Table 3:** Number of appointments offered by EDYS each month from December 2019 to July 2020 split by appointment type

2019–2020	Face-to-face (n)	Virtual (n)	Both (n)
December	222	0	222
January	288	0	288
February	285	0	285
March	Data by mode of apt not available		361
April	66	352	418
May	88	277	365
June	135	285	420
July	219	229	448
Total			2807

### Perceptions of how young people with eating disorders are coping in COVID-19: patient, parent/carer and staff perspectives

**Fig. 1:**
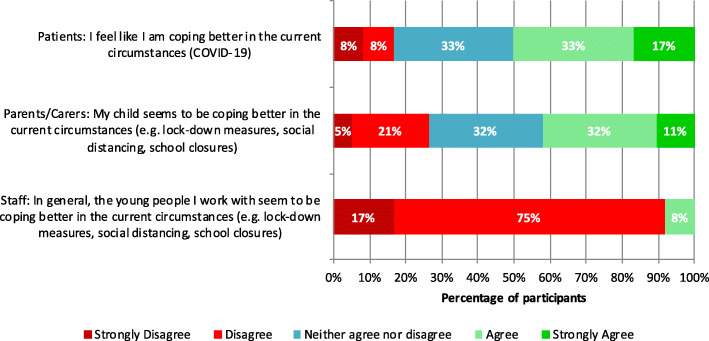
Percentage of agreement (strongly disagree [1] to strongly agree [5]) for patients, parents/carers and staff on how young people with eating disorders are coping in COVID-19

Figure 1 shows the percentage of agreement (strongly disagree [1] to strongly agree [5]) for patient, parents/carers and staff when asked how young people with eating disorders were coping in COVID-19. Views differed between groups, with 92% of staff disagreeing that young people were coping better, 26% of parents/carers and only 16% of patients.

### Self-reported types of appointments experienced in COVID-19 and staff, patient and parent/carer preferences

**Fig. 2:**
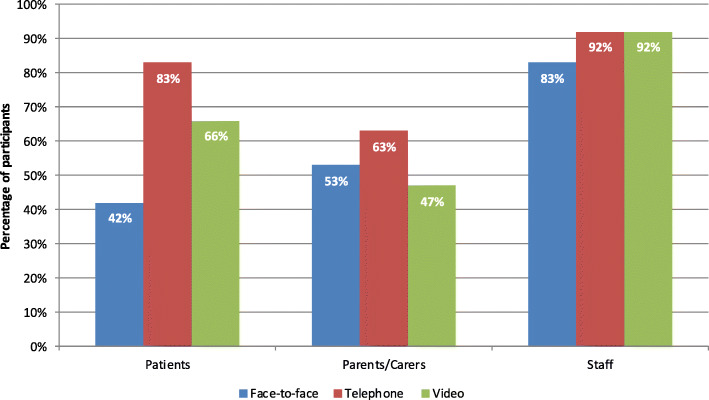
Types of appointments experienced during COVID-19: self-reported by patients, parents/carers and staff

**Fig. 3:**
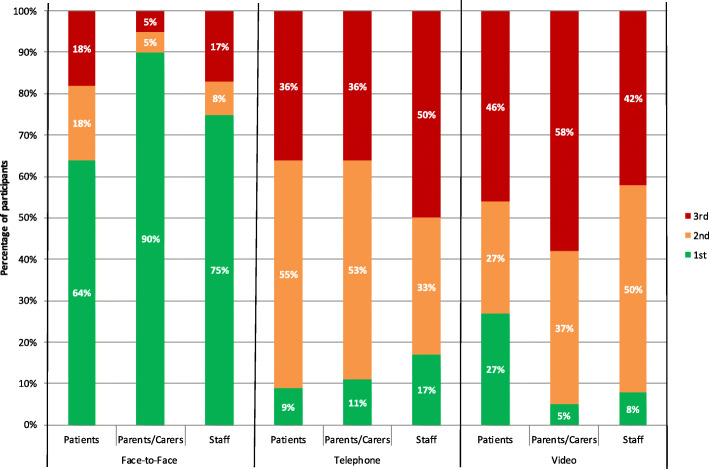
Patient, parent/carer and staff ratings (1st, 2nd or 3rd) of types of appointment based on preference

Most staff reported experiencing the full range of appointments but patient and parents were more likely to experience remote contact from the service (Fig. 2). Of the types of contact offered, face-to-face was the preferred type of appointment for staff, patients and parents/carers (Fig. 3). Preferences varied within groups and more patients put video calls as their first choice compared to staff and parents/carers.

### Service satisfaction before and during COVID-19: a comparison

Patients satisfaction with service slightly increased during COVID-19, whilst parents/carers and staff’s slightly decreased (Table 4). A Wilcoxon Signed-rank test indicated that there was no significant difference between service satisfaction before COVID-19 (Mdn = 4) and during COVID-19 (Mdn = 4), T = 26, *p* = .14, r = .43.

**Table 4.** Median Scores of patient, parent/carer and staff service satisfaction before COVID-19 and during COVID-19

Topic of Item	Patient		Parents & Carers		Staff		All participants	
	Before	During	Before	During	Before	During	Before	During
The help provided in general	4	4.5	5	5	4	4	5	4
Information provided about the service and help available	4	4.5	4.5	4	4	4	4	4
Accessibility of appointments/groups	5	4	4.5	4	4	3.5	4	4
Ease of contacting the team	4	4.5	4.5	4	4	4	4	4
Enough going on for the young person to get involved in	4	4	4.5	4	4	2.5	4	4
**Total**	4	4.5	4.5	4	4	4	4	4

### Qualitative

Table 5 summarises the key changes described by staff, patients and parent/carers.

**Table 5:** Summary of Key Changes

Changes Described	Staff experience	Patient’s experience	Parent’s/Carer’s experience
Experiencing “lockdown” restrictions.	Having one clinician onsite- difficult to fit face to face contacts in. Lonely, isolating. Missed colleagues support.	Have more space and time to focus on recovery. Feel like missing out on less in general life. Relief avoiding restaurants. Less distractions, more time to think. More exposure to social media.	Fantastic team response, always available and responsive despite lockdown.
Moving to virtual video consultations.	Feels inappropriate for high risk. Those in good recovery engaged well. Not limited by room availability.	Travel avoided which can be tiring. Easier to access from home. Harder to open up on a screen. Can’t see body language. Feels easier to hide how actually doing. Feels less genuine. Technological difficulties are a barrier. Miss face-to-face as easier to open up. Would enjoy choice but may choose easier option for anorexia.	Mix would be good to limit demands of travel. Difficult when IT and connection problems. Face-to-face needed to build trust in beginning. Affected discharge and ending – felt abrupt, left with high anxiety.
Having telephone appointments.	Feels inappropriate for high risk cases. Less effective. Raised staff anxieties.	Felt family were less involved and received less support. Shorter consultations. Need family to be involved and a bigger part of it.	Impractical. Family sessions don’t involve family. Sessions much shorter. Feel left on own with anxieties. Feel more responsibility to communicate what is not visible e.g., child’s response.
Using emails for contact.		Enjoyed being able to ensure messages got to practitioners and enjoyed quick response.	
Being restricted to one parent in the session.	Asking one parent to relay information to another parent not effective. Need whole family for family treatment.	Wished parents had more support. Parents need family sessions for themselves and to help recovery. Parents essential to recovery.	
Accessing/Offering support from home environment.	Difficult. Much rather be on site. Isolating. Can be left with strong feelings on own, at home. Very hard managing childcare at the same time. Miss travel to switch off.	More comfortable. Easier to leave if want to. Less afraid of practitioners and consequences of not adhering to meal plan. Feel intimidated to ask for something else as feels like how the service is provided is not within the services control.	Able to give more support to child, more supervision. Better meet their needs. Feels more intense in lockdown. No respite. Harder if can’t get food in shops. More to deal with.
Virtual MDTs.	Good to have more space on own, not limited by room size. Easier to access and attend. IT issues make more challenges. Not seeing people’s faces hard to pick up cues. Affects feeling of team working. Easily distracted by laptops.		

### Positive experiences

#### Continuing to provide a fantastic service

Parents and patients reported that they appreciated having a responsive, supportive multidisciplinary team that remained available. Those that received face-to-face appointments were grateful that these remained available, despite lockdown pressures. Feedback from parents stated that the service maintained integrity despite the challenges. Staff were, however, less likely to comment on and acknowledge their successes.

### Improving access

Using virtual platforms improved ease of access. Staff reported working with more autonomy, and offering more contact as they were not constrained by room availability on the hospital site. Staff felt more able to increase the number of contacts they had within the day as they had more flexibility and a variety of mediums to choose from. Patients reported enjoying using emails to contact staff, as they felt confident they received the message, and received a prompt reply. Staff reported that that their knowledge of online support available improved. Some parents reported finding it easier to meet their child’s emotional needs while they were at home together. Staff reported that virtual MDTs meant they could join from anywhere and have more space in the room. Staff also described a benefit of video sessions being that they could share online resources easily.

### Increasing comfort

Patients and family members reported that having a mixture of video and telephone contact reduced travel costs and left them feeling less drained. Patients commented that they also felt less tension and “build up” attending appointments. Some staff felt that having contact within their home improved relationships as people seemed to open up more within a comfortable home environment. Although some patients found it easier to open up when they were not face to face, the majority described the opposite. Patients reported enjoying a break from the pressures of “normal” life, e.g., visiting restaurants.

### Negative experiences

#### Increased isolation and fragmentation

Family members were less likely to attend appointments with the young person which all participants reported as a major issue with lockdown restrictions. FBT did not lend itself to virtual or telephone therapy as it was hard to include everybody in the process. Endings felt more abrupt, incomplete and individuals were left feeling more anxious about relapse. Staff also reported reduced capacity to monitor physical health in the community as other sites were not in full operation. At the beginning, there was only one practitioner on site which practitioners found very “lonely”. Practitioners said they found it harder to make decisions about risk, or care plans, when there was nobody else on site. Staff that remained working from home described being left with “strong feelings” at the end of a difficult day, which could leave them feeling stressed in a place they could usually relax.

### Altered relational experiences

The restrictions changed the experience of relationships and contacts. Staff and patients reported finding it difficult to wear PPE and talk in face-to-face sessions. Patient reported preferring not to wear PPE, and finding the video sessions easier as they did not have to talk through a mask.

Using technology impacted on the quality of experience within services. Staff, patients and parents/carers all preferred having some face-to-face contact. Patients found it frustrating if they experienced technological difficulties whilst sharing their feelings as it was difficult enough to “open up” and “share”. Staff found it hard not having access to a printer to give out material for people to take away. Patients said that in some ways the video sessions felt “less real”, and that they also felt under less pressure from services if they were not being seen. Patients reported feeling like they could easily get out of the situation if they wanted to by hanging up. This was positive in some ways, but others reported that they needed a “check in” and “eyes on” to resist the demands of anorexia. Some patients described feeling like they needed to be “uncomfortable to get comfortable again.” Whilst patients may have opted *not* to come in if they had a choice, they acknowledged that it was necessary to experience the feelings they did in sessions to make a full recovery.

Families and patients reported that telephone consultations were more likely to be shorter. All participants felt telephone sessions were least effective. Staff commented that having face-to-face sessions was vital at the beginning of a patient’s journey, when individuals needed more intensive input. Patients described struggling to open up with people they first met online, as it was hard to trust someone without meeting them. Patients and parents described feeling like it was not always clear if they were allowed face-to-face contact, or not. Patients said they would avoid asking for something different to what was offered as they did not want to put pressure on the NHS.

Some staff found that working virtually affected their role and made the work less satisfying. Others reported that it affected team support and morale. For a new member joining the team, it was a harder experience being inducted and understanding processes. Virtual MDTs could go on for longer as it was harder to read the cues of attendees and know when to stop. Virtual MDTs were described as being more task orientated, although staff commented this was likely impacted by increased service pressures too.

### Increased acuity and more pressure

Parents reported finding it more intense offering support to their child whilst working from home, and without the support from school. Staff were generally much more likely to comment on the increased pressure on the service. Staff reported having more presenting risk in the cases that were referred during lockdown. They also report missing the travel time to “switch off” and have a break from work. Many staff reported working longer hours from home and over working. Staff talked about increased pressure managing their home life during a pandemic, in particular, having to care for their own children from home whilst providing a clinical service. One staff member reported that the childcare provision during the pandemic did not align with the hours of service provision.

Patients felt it was unfair that “pubs could open” but mental health services were not prioritised. Patients and their parents described struggling with a lack of routine and structure. Patients also said they found they were more likely to spend time on their phones and be exposed to social media which hindered their recovery.

## Discussion

This is the first mixed methods study exploring how patients, families and staff are experiencing the new ways of working driven by COVID-19 in a young person’s eating disorder service. Although there was no difference in service satisfaction before COVID-19 and during COVID-19, face-to-face was the preferred type of appointment for patients, parents/carers and staff.

In our focus groups, we observed a marked difference in the views of staff, parents and patients. Staff and parents have different insights into a child’s experience of treatment in an eating disorder service. Staff are more likely to have experienced the service prior to lockdown and may be judging and evaluating the service based on how it has run before. Parents may be experiencing the service for the first time and are likely grateful for the support from any service during lockdown as many healthcare providers stopped offering treatments. It is also likely that the parents that took part in the focus group were more likely to be engaging well with the service, and virtual treatments, as they provided feedback through virtual platforms. However, staff saw a broader spectrum of presentations, and were able to comment on the challenges they faced with engagement in treatment and risks that were difficult to manage. Staff also faced different challenges in running a service, which children and families were unaware of. It could be that this stress and pressure influenced evaluations of the service.

Despite inviting all patients open to the service and their parents/carers, the small sample size is an important limitation of the study that must be noted. As the study intended to capture the impact of a global pandemic lockdown period on experiences within an eating disorder service, a change in government restrictions meant we were unable to extend the recruitment window. As restrictions lifted, changes to service provision were reversed and face-to-face appointments were no longer limited to high-risk patients. Given the small sample size, the conclusions of this paper should be treated with caution and further research should be conducted to validate the findings. Future research should be broken down into diagnostic groups (e.g. AN, BN and BED) so that differences can be reported and discussed. This is another limitation of the current study, but as diagnostic labels are not always used in treatment at this service, it was felt it would have been inappropriate to ask patients and parents/carers this as part of the survey. As the caseload of patients registered with EDYS is relatively small, it is common for there to be only one patient of a certain age or gender at one point in time. Therefore, collecting basic demographics could have violated ethical approval, as clinicians analysing the data may have been able to identify which patient had submitted the response. However, including this information would add value to future research. We also recognise there is likely to be a self-selection bias with regards to the sample, as those who are in the later stages of treatment are more likely to have the time and motivation to respond. However, staff will be seeing patients at all stages of their recovery and their answers reflect this. This may explain the disparity between staff, parent’s and patient’s perceptions of how young people are coping in lockdown. The key changes to service provision were centred on the restriction measures implemented by the government and the use of virtual services to provide treatment. Below we will outline the key themes identified before making clinical recommendations to guide service delivery.

### Locking down but ramping up

Despite being forced to minimise direct patient contact, the service saw an increase in inpatient admissions and referrals, during and following lockdown. This implies that the pandemic is not only having a negative impact on those who already have an eating disorder as suggested by Branley-Bell and Talbot [2], but is also increasing the likelihood of young people developing an ED. Patients reported having less distractions and more exposure to social media was a negative consequence of the lockdown restrictions on recovery; which is commensurate with other recent research [2, 6]. Parents/carers expressed concerns about obtaining food from the shops and described the experience of caring for a child with an eating disorder as more intense. All participants described a frustration with the government guidelines. In particular, patients felt it was unfair that “pubs could open” but mental health services widely could not. Despite limitations, there was a considerable increase in the number of appointments offered by EDYS during lockdown in comparison to the previous months. Staff reported that they had more flexibility to offer appointments with the new ways of working, but also reported overworking and finding it harder to manage risk. Staff commented on the difficulties of limiting service provision, whilst dealing with more acute presentations. At a time of increased need, and more presenting risk, service delivery was compromised.

### The Silver lining

Online/virtual treatments were recommended to reduce the risk of viral transmission [6] and as a result, EDYS had to quickly adopt remote consultations. Only 42% of patients that took the survey had experienced face-to-face appointments. Despite most individuals favouring face-to-face contact, having the flexibility to use different mediums improves ease and access. Appointments would no longer be limited by room availability or parent/carer availability. Attending appointments from home can also reduce treatment burden through reducing travel and limiting the associated costs (e.g. public transport fares, petrol, and parking). Furthermore, using emails would not previously have been part of practice, but was greatly valued by patients. Staff reported an improved knowledge of online support available for the young people they were seeing, which may continue to benefit current and future patients. Service satisfaction did not change during COVID-19 despite the significant changes to service provision.

### Timing is everything

Given the evidence for intensive interventions at the early stages of an ED [5, 8], it is unsurprising that all participants felt face-to-face contact was crucial at the beginning of treatment. Staff felt that virtual appointments were inappropriate for high risk patients given the difficulty of adequately assessing physical and mental health risks remotely [12]. In line with this, patients felt they needed the face-to-face contact to resist the demands of their ED. At the right time, virtual appointments can be effective and act as a step down. ED services must carefully assess when it is appropriate to start using virtual interventions for their patients, on a case to case basis. Introducing virtual methods when they are deemed as effective can also increase availability of rooms for the cases where face-to-face contact is vital. It should also be considered, that the absence of face-to-face appointments before discharge, left parents feeling like endings were abrupt, leaving them with more anxieties about the cease in support. Careful consideration of the implementation of virtual methods will maximise the chance of them providing a positive addition.

### Learning from the losses

A lack of time to prepare, made changes to services more challenging. In this sample, patients and their carers felt there was a lack of support for whole families during restrictions. Given that FBT is advocated as the evidence-based intervention for child and adolescent eating disorders [15], it is important that services ensure that the family are not forgotten. As it was generally felt that FBT was less effective virtually, careful planning must go into how services can involve and support families moving forward. Despite lockdown measures easing, staff reported still being restricted to one parent per session. A balance of risks should be considered as families are within the same “bubble” and pose limited additional risk, whereas the reduction in treatment effectiveness could be life threatening. Future research must aim to understand how best to adapt treatments, such as FBT, for virtual delivery to ensure that it is safe and effective [10].

Over time, access to technology has improved but many individuals still struggle to navigate this without guidance. All participants reported issues with connectivity, which had a negative impact on engagement in sessions. As virtual interventions are also only accessible to patients and families who have the relevant devices, there is a risk of services becoming less inclusive. Being aware of the barriers to accessing services virtually and at home is of paramount importance.

Restrictions also involved limiting the number of clinicians allowed onsite, leaving staff feeling lonely and isolated, which has also been found in other studies [4, 14]. Staff reported missing the support of their colleagues and were left with strong feelings to manage alone; whether that be in the hospital or at home. Staff found it harder to make decisions about risk or care plans when there was nobody present to discuss this with. This must be considered alongside other challenges of working remotely, such as the ability to switch off and stay well. While the service kept running and remained effective, we must wonder at what cost? Staff were less likely to comment on their successes and described working extra hours, whilst struggling to manage caring commitments at home. The negative psychological effects of the pandemic include higher rates of burnout, PTSD, anxiety and depression in NHS staff [9]. It is now even more important to look after the health and wellbeing of NHS Staff, as called for in the NHS People Plan (2020–2021).

## Conclusions

The Covid-19 global pandemic and the associated lockdown restrictions increased the pressure on a young person’s specialist eating disorder service in the UK. The service saw more acute presentations in those referred and more referrals following the lockdown. The elevated levels of risk felt harder to manage with restrictions to service provision. Despite the significant changes to service delivery, an eating disorder service that children and parents report high satisfaction with can still be offered during lockdown restrictions. Email contact with practitioners improved responsiveness and was perceived as a positive change. Virtual and telephone appointments increased flexibility within the service and improved autonomy for practitioners. However, the quality of the relational experience is altered online and through virtual mediums. In the context of appointment types, patient choice must be carefully considered, as individuals may want to resist pressure and providing face-to-face appointments is necessary in the treatment of acute anorexia. Including parents/carers in treatment plans is essential and appointments with multiple people in a room need to be prioritised for family-based treatments. A mixture of appointment types is likely to be the most effective in the treatment of eating disorders in young people. Those providing services may face new and intense pressures, putting practitioners at an increased risk of burnout. Multiple practitioners on site are required to support decision making about risk and to share ideas to deliver MDT working. Supporting NHS staff is a vital part of continuing to deliver high quality NHS services.

## Data Availability

The datasets generated during and/or analysed during the current study are available from the corresponding author on reasonable request.

## Abbreviations

ED: Eating Disorders
EDYS: Eating Disorder Young Person’s Service
FBT: Family-based Therapy
ESQ: Experience of Service Questionnaire
CAMHS: Child and Adolescent Mental Health Service
